# The Role of cAMP in Beta Cell Stimulus–Secretion and Intercellular Coupling

**DOI:** 10.3390/cells10071658

**Published:** 2021-07-01

**Authors:** Andraž Stožer, Eva Paradiž Leitgeb, Viljem Pohorec, Jurij Dolenšek, Lidija Križančić Bombek, Marko Gosak, Maša Skelin Klemen

**Affiliations:** 1 Institute of Physiology, Faculty of Medicine, University of Maribor, SI-2000 Maribor, Slovenia; andraz.stozer@um.si (A.S.); eva.paradiz1@um.si (E.P.L.); viljem.pohorec@um.si (V.P.); jurij.dolensek@um.si (J.D.); lidija.bombek@um.si (L.K.B.); marko.gosak@um.si (M.G.); 2 Faculty of Natural Sciences and Mathematics, University of Maribor, SI-2000 Maribor, Slovenia

**Keywords:** cAMP, beta cells, stimulus–secretion coupling, intercellular coupling, PKA, Epac2A

## Abstract

Pancreatic beta cells secrete insulin in response to stimulation with glucose and other nutrients, and impaired insulin secretion plays a central role in development of diabetes mellitus. Pharmacological management of diabetes includes various antidiabetic drugs, including incretins. The incretin hormones, glucagon-like peptide-1 and gastric inhibitory polypeptide, potentiate glucose-stimulated insulin secretion by binding to G protein-coupled receptors, resulting in stimulation of adenylate cyclase and production of the secondary messenger cAMP, which exerts its intracellular effects through activation of protein kinase A or the guanine nucleotide exchange protein 2A. The molecular mechanisms behind these two downstream signaling arms are still not fully elucidated and involve many steps in the stimulus–secretion coupling cascade, ranging from the proximal regulation of ion channel activity to the central Ca^2+^ signal and the most distal exocytosis. In addition to modifying intracellular coupling, the effect of cAMP on insulin secretion could also be at least partly explained by the impact on intercellular coupling. In this review, we systematically describe the possible roles of cAMP at these intra- and inter-cellular signaling nodes, keeping in mind the relevance for the whole organism and translation to humans.

## 1. Introduction

**Figure 1:**
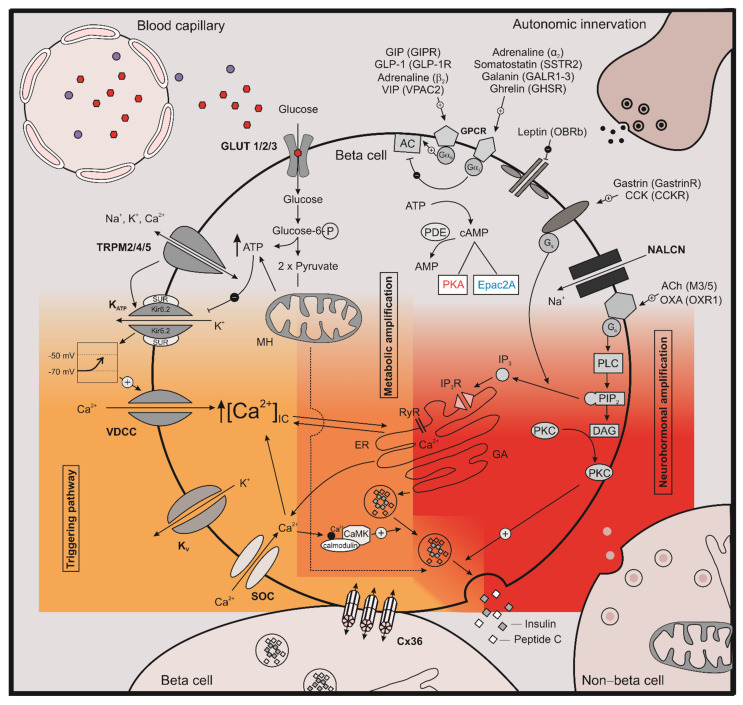
Stimulus–secretion coupling in beta cells. The three interconnected intracellular signaling pathways in pancreatic beta cells are shown; the K_ATP_ dependent triggering pathways is indicated in dark yellow, the metabolic amplifying pathway in orange, and the neurohormonal amplifying pathway in red. Parts of a non-beta cell and a neighboring Cx36-connected beta cell are shown on the bottom, blood capillary and autonomic innervation are shown on top. GLP-1—glucagon-like peptide 1; GIP—glucose-dependent insulinotropic peptide; GIPR—glucose-dependent insulinotropic peptide receptor; GLP-1R—glucagon-like peptide 1 receptor; b2—beta-2 adrenergic receptor; a2—alpha-2 adrenergic receptor, VPAC2—vasoactive intestinal peptide receptor 2; GALR1-3—galanin receptor 1-3; SSTR2—somatostatin receptor 2; GHSR—growth hormone secretagogue receptor; OCRb—long form of the leptin receptor; M3-5—muscarinic acetylcholine receptor 3-5; OXA—orexin A; OXR1—Orexin-1 receptor; GPCR—G-protein-coupled receptor; cAMP—cyclic adenosine monophosphate; AMP—adenosine monophosphate, PDE—phosphodiesterase; PKA—protein kinase A; Epac2A—exchange protein directly activated by cAMP; TRPM—transient receptor potential melastatin; K_ATP_—ATP dependent potassium channel; Kir6.2—major subunit of the ATP-depedent K^+^ channel; SUR—sulfonylurea receptor; VDCC—voltage-dependent calcium channel; K_v_—voltage-dependent potassium channels; SOC—Store-operated channel; MH—mitochindrion; AC—adenylyl cyclase, ER—endoplasmic reticulum; VIP—vasoactive intestinal polypeptide; ACh—acetylcholine, PLC—phospholipase C; PIP_2_—phosphatidylinositol 4,5-bisphosphate; IP_3_—inositol trisphosphate; IP_3_R—inositol trisphosphate receptor, DAG—diacylglycerol; PKC—protein kinase C; GA—Golgi apparatus; K^+^—potassium ions; Na^+^—sodium ions; Ca^2+^—calcium ions; RyR—ryanodine receptors; Cx36—connexin-36; CAMK—Ca^2+^/calmodulin-dependent protein kinase, glucose-6P—glucose-6 phosphate; [Ca^2+^]_IC_—intracellular calcium concentration; G_q—_Gq protein alpha subunit; Gas—Gs alpha subunit; Gai—Gi protein alpha subunit; NALCN—sodium leak channel; CCK—cholecystokinin; CCKR—cholecystokinin receptor.

Insulin secreted by pancreatic beta cells regulates storage and usage of nutrients, and its relative or absolute lack results in diabetes mellitus that affects more than 460 million people around the world, a number that is expected to increase to 700 million by 2045 [1]. If we consider also undiagnosed diabetes, the picture is even worse. The disease burden is immense and includes both public and personal costs [2], with total annual global health expenditures for diabetes estimated to more than 760 billion USD [1]. More than 95% of all people with diabetes have type 2 diabetes mellitus (T2DM), which is characterized by obesity, insulin resistance, and insufficient insulin secretion [3]. Numerous antidiabetic drugs with different molecular mechanisms are available for T2DM treatment, among which sulfonylureas (SUs) are the most widely prescribed. They are very potent antidiabetic drugs, sufficient to activate the so-called triggering pathway, but bear a risk of hypoglycemia and weight gain [4]. The triggering pathway of insulin secretion in beta cells consists of several steps (Figure 1), starting with the influx of glucose through glucose transporters (GLUT), glucose metabolism increasing cytosolic adenosine-triphosphate (ATP) concentration, and the subsequent closure of ATP-dependent K^+^ (K_ATP_) channels. The decrease in K^+^ efflux causes membrane depolarization, followed by the opening of voltage-dependent Ca^2+^ (VDCC) channels and the influx of Ca^2+^ ions. The resulting increase in the intracellular concentration of Ca^2+^ ([Ca^2+^]_IC_) triggers insulin secretion by activating the secretory machinery and fusion of insulin-containing vesicles with the plasma membrane [5,6,7]. SUs stimulate insulin secretion by closing K_ATP_ channels by binding to their SU receptor 1 (SUR1) [8,9,10,11]. Besides the risk of hypoglycemia, chronic treatment with certain SUs could lead to the progressive failure of beta cells, since tolbutamide and glibenclamide were found to induce beta cell apoptosis in rat [12] and human islets [13].

Beside SUs, the incretin-related drugs are increasingly being used for stimulation of insulin secretion. The incretin hormones secreted from the gut augment insulin secretion observed after an oral glucose intake compared with that observed after identical elevation of plasma glucose with a controlled intravenous infusion of glucose [14,15,16,17,18,19,20]. Among incretins, glucose-dependent insulinotropic polypeptide (GIP) and glucagon-like peptide-1 (GLP-1) are the most investigated [21]. GIP and GLP-1 are released into the bloodstream from enteroendocrine K and L cells, respectively, following meal ingestion [22,23]. In pancreatic beta cells, GIP and GLP-1 bind to the specific guanine nucleotide-binding protein-coupled receptors (GPCR) GIPR and GLP-1R, respectively [24,25,26]. While, in healthy humans, oral administration of glucose triggers higher insulin secretion than a comparable glucose challenge intravenously due to the incretin effect, in T2DM, this phenomenon is partly lost, but the attenuated insulinotropic action is observed only for GIP. On the other hand, the actions of GLP-1 remain relatively preserved, although the levels of GLP-1 are significantly decreased [27,28,29]. Therefore, therapeutic approaches are mainly oriented at enhancing GLP-1 action through degradation-resistant GLP-1R agonists (incretin mimetics) or through inhibitors of the enzyme dipeptidyl-peptidase-4, which is responsible for a rapid degradation of incretin hormones. The effect of GLP-1 administration in humans was described over 20 years ago [30], leading to development of GLP-1 based therapies for T2DM [31], and improved drugs with GLP-1R agonist activity are currently being developed [32]. In mice, GLP-1 or GLP1-R agonists clearly improve glucose tolerance and increase glucose stimulated insulin secretion (GSIS), their effects ranging from normal mice [32] to the alloxan-induced diabetic model [33]. Accordingly, the oral glucose tolerance test (OGTT), known to elicit release of incretins, results in larger increase of blood glucose levels in GLP1-R knock-out (KO) mice compared with wild-type mice, corroborating the importance of GLP-1R signaling for normal glucose tolerance [34]. Noteworthy, GLP-1R KO mice are glucose-intolerant also in intraperitoneal glucose tolerance tests (ipGTTs), in which the level of incretins is not increased. Therefore, it seems that even the basal level of GLP-1 or some constitutive activity of the GLP-1R signaling pathway is important for normal glucose tolerance. Furthermore, inhibition of GLP-1R with an antagonist Exendine-9 reduces the insulin response to oral glucose by 30–40% [35] but with 1.5- to 2-fold variability among nondiabetic subjects [36], which points to differences in the β-cell sensitivity to GLP-1. The GLP-1R sensitivity can be calculated from the insulin secretion rate using a progressive increase in plasma GLP-1 via infusion during a hyperglycemic clamp. In nondiabetic human subjects, the obese individuals who are more insulin resistant showed increased slope of insulin secretion rate in response to stepwise elevation of GLP-1 compared to lean individuals [37]. Sensitivity of insulin secretion to GLP-1 is clinically important because of the reduced incretin effect in T2DM patients [38], a finding recently proposed to extend to obese subjects [37,39]. Since incretins have almost no effect on insulin secretion in low glucose [40,41], this makes them a much safer option in terms of risk of hypoglycemia compared with SUs. In addition to the established effects on beta cell insulin secretion, GLP-1 also seems to induce a paracrine beta cell response, which leads to alpha cell proglucagon processing that results in GLP-1 production. This process is not yet fully understood; however, it may be metabolically relevant [42].

In pancreatic beta cells, GLP-1 and GIP exhibit their effect through the so-called neurohormonal amplifying pathway (Figure 1). Binding to GPCR results in interaction with the Gαs subunit, with subsequent activation of adenylyl cyclase and consecutive production of 3′,5′-cyclic adenosine monophosphate (cAMP) from ATP. In beta cells, cAMP is one of the most important cellular signaling molecules that amplifies insulin secretion elicited by the triggering signal. On the other hand, degradation of cAMP to 5′-AMP is regulated by phosphodiesterases (PDEs), and cAMP lowering agents thus inhibit insulin secretion (Figure 1). It was found that cAMP signaling in not only triggered by GPCR agonists but also under glucose stimulation promoting cAMP elevation with pronounced oscillations. This Ca^2+^-dependent mechanism activates certain adenylyl cyclases (AC) and is important mainly at substimulatory glucose concentration, while, under increased glucose concentration, a metabolic amplification of cAMP also becomes important [43]. The actions of cAMP are further mediated via protein kinase A (PKA) and the guanine nucleotide exchange factor Epac2A. It was also shown recently that PKA isoform with Riα (PKA-Riα) could act independently of the cAMP signaling mechanism [44]. On the other hand, Epac2A is also directly activated by various SUs, except for gliclazide, and activation of Epac2A signaling seems to be required for SU-induced insulin secretion [33,45,46]. Furthermore, binding properties of various SUs to Epac2A were quantified, and it was found that cAMP and SUs cooperatively activate Epac2A [47,48]. This molecular mechanism provides a possible explanation for additive effects of combination therapies of incretin-related drugs and SUs, although many important questions still need to be answered in this regard.

Since the incretin effect in pancreatic beta cells is mediated via the neurohormonal amplifying pathway through PKA and Epac2A, the specific role of PKA in glucose homeostasis was assessed in vivo by disinhibiting PKA activity in a genetic mouse model [49]. A conditional homozygous ablation of the PKA regulatory subunit (Prkar1a KO) resulted in increased glucose tolerance with a concomitantly enhanced GSIS after ipGTT. The enhancing role of PKA was confirmed also in humans in the same study [49]. When crossing Prkar1a KO mice with Epac2A KO mice, the resultant Prkar1/Epac2A KO mice exhibited a reduction in GSIS compared with Prkar1a KOs [50]. This implicates that Epac2A expression is permissive and necessary for the maximum effect of GLP-1R stimulation on GSIS.

Regarding the role of Epac2A, several studies demonstrated that Epac2A ablation failed to exert any influence either on glucose tolerance or on GSIS after ipGTT in mice [46,50,51]. Interestingly, Epac2A KOs exhibit early morphological (e.g., increased fat deposits) and biochemical (e.g., increased leptin and decreased adiponectin serum levels) signs of obesity, but these changes are connected with suppressed hypothalamic leptin signaling [51]. On the other hand, when beta cell insulin secretion was potentiated pharmacologically by GLP-1R activators, the neurohormonal amplifying pathway was activated, and Epac2A KO mice revealed an impaired glucose tolerance and GSIS during ipGTT [50]. This suggests that Epac2A plays a crucial role in mediating the effects of incretins on GSIS rather than contributing to GSIS without additional stimulation by incretins. Surprisingly, the same study reported that, during OGTT, glucose tolerance was not perturbed in Epac2A KO mice [50]. It is possible that Epac2A plays a role in increasing GSIS at pharmacological levels of GLP-1-R stimulation but not at more physiological concentrations of GLP-1 reached during an OGTT. Furthermore, Epac2A seems to be important also during the development of insulin resistance and in combination therapies for T2DM treatment involving incretins and SUs. In sum, the role of GLP-1R signaling in maintaining normal glucose tolerance in general and the differential roles of Epac2A and PKA arms of this neurohormonal amplifying pathway in specific await further clarification.

Beta cells in the pancreatic islets display coordinated insulin secretion. This is a result of different means of intercellular communication, which ensures that heterogeneous beta cell populations work in synchrony [52,53,54]. Gap junctions composed of connexin36 (Cx36) are the key synchronizing agents that coordinate electrical activity across the islet in the form of intercellular waves [55,56,57,58]. Recent research suggests that impaired gap junction coupling is associated with disruptions to glucose homeostasis, altered islet function, and impaired plasma insulin oscillations, similar to that observed in models of diabetes [59,60,61,62,63]. In this vein, cell-to-cell communication was recognized as one of the underlying factors which contributes to the pathogenesis of diabetes [64,65]. Furthermore, intercellular coupling in islets as well as in other systems was also reported to be modulated by cAMP signaling, but the findings about the effects of PKA-dependent and PKA-independent mechanism involving Epac2A are rather diverse [66,67,68,69,70,71,72,73,74]. This underlines the necessity for further studies on how the neurohormonal signaling pathways influence gap-junctional communication and the collective rhythmic activity in islets, as the effect of cAMP on insulin secretion could also be at least partly explained by the impact on intercellular coupling.

Taken together, PKA and Epac2A may play an important role in (ⅰ) normal glucose homeostasis, (ⅱ) incretin potentiation of GSIS, (ⅲ) potentiation of GSIS in obesity, and (ⅳ) potentiation of GSIS in combination therapy involving SUs and incretins. The molecular mechanisms behind this are still not fully elucidated and involve many steps in stimulus–secretion coupling (SSC), ranging from the regulation of ion channel activity and the triggering Ca^2+^ signal to the most distant step in SSC, exocytosis. Finally, the effect of cAMP on insulin secretion could at least partly be explained also by the impact on intercellular coupling. In the subsequent chapters of this review, we focus on these intra- and inter-cellular mechanisms in more detail.

## 2. The Role of cAMP on Stimulus Secretion Coupling

### 2.1. The Effect of cAMP on Ion Channels

Ni et al. found that oscillations in PKA activity directly mirror the oscillations in cAMP levels [75]. The cAMP-mediated potentiation of insulin secretion is further regulated by (ⅰ) activators of AC and (ⅱ) inhibitors of PDEs, which degrade cAMP, and (ⅲ) is stimulated by high [Ca^2+^]_IC_ [76].

**Figure 2:**
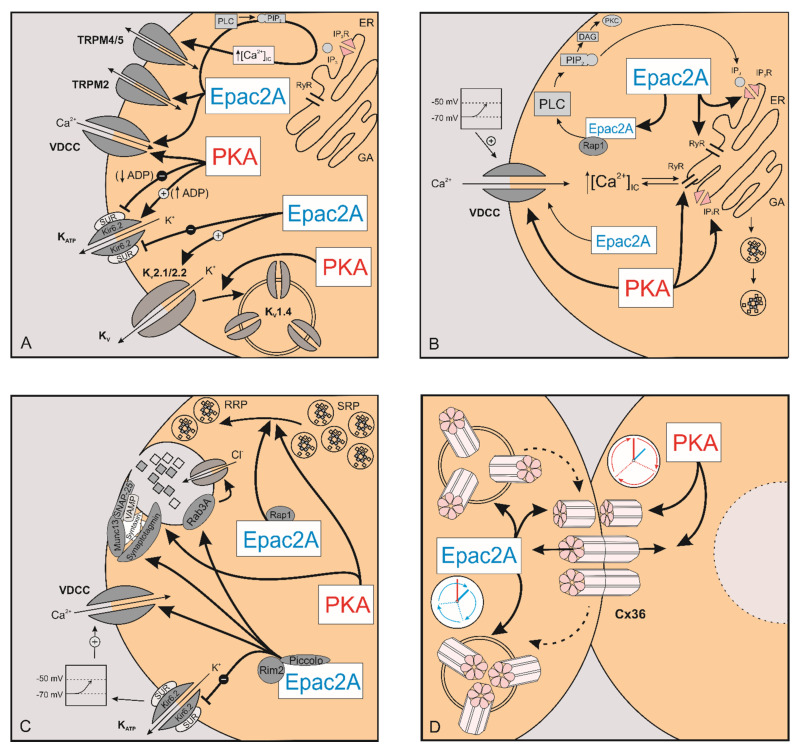
The role of PKA and Epac2A in stimulus–secretion and intercellular coupling. The effect of PKA and Epac2A on ion channels (**A**), [Ca^2+^]_IC_ (**B**), exocytosis (**C**), and intercellular coupling (**D**). PKA—protein kinase A; Epac2A—exchange protein directly activated by cAMP; TRPM2—transient receptor potential melastatin 2; TRPM4/5—transient receptor potential melastatin 4/5; ADP—enosine diphosphate; K_ATP_—ATP dependent potassium channel; Kir6.2—major subunit of the ATP-depedent K^+^ channel; SUR—sulfonylurea receptor; VDCC—voltage-dependent calcium channel; K_v_—voltage-dependent potassium channels; ER—endoplasmic reticulum; PLC—phospholipase C; PIP_2_—phosphatidylinositol 4,5-bisphosphate; IP_3_—inositol trisphosphate; IP_3_R—inositol trisphosphate receptor, DAG—diacylglycerol; PKC—protein kinase C; GA—Golgi apparatus; K^+^—potassium ions; Na^+^—sodium ions; Ca^2+^—calcium ions; Cl^−^—chloride ions; RyR—ryanodine receptor; Cx36—connexin-36; [Ca^2+^]_IC_—intracellular calcium concentration; Rim2—Rab GTPase interacting molecule; Rpa1—Ras-related protein 1; Munc13—mammalian uncoordinated-13; VAMP—vesicle-associated membrane protein; Rab3A—Ras-related protein Rab-3A; SNAP-25—synaptosomal-associated protein 25; RRP—readily-releasable pool; SRP—slowly releasable pool.

Since AC is a transmembrane protein, the highest cAMP concentration can be expected to be at the submembrane compartment, which is of great relevance for the regulation of ion channels (Figure 2A) and for the process of exocytosis [43]. Moreover, research shows that submembrane cAMP levels vary locally to a great extent. This is attributed to A-kinase anchoring proteins (AKAPs) which scaffold AC of different isoforms, PKA, GPCR, PDE, and other pertaining signaling components in cAMP production into very confined compartments to generate local ATP pools [77]. This clustering of Ca^2+^-sensitive AC into nanodomains is then responsible for local increases in cAMP levels, which are exactly in-phase with Ca^2+^ oscillations, whereas localized PDE is responsible for the out-of-phase decreases in local cAMP [78].

#### 2.1.1. KATP Channels

K_ATP_ channels belong to a family of inward-rectifying K^+^ channels [79]. They are composed of four potassium-selective pore-forming Kir6.2 subunits which have binding sites for ATP and four regulatory SUR1 subunits with binding sites for MgADP, enabling them to be controlled by changes in intracellular ATP concentration ([ATP]_IC_) and the ratio of intracellular ATP to adenosin diphosphate ([ADP_IC_]) [ATP]_IC_/[ADP]_IC_ [80,81].

Light et al. demonstrated that PKA regulates K_ATP_ channels in an ADP-dependent manner [82]. When glucose concentration is high, the cytoplasmic ADP levels are relatively low (ATP production is high), and PKA inhibits K_ATP_ channels, while, at elevated ADP levels (low glucose), it increases their activity. As proposed by some authors, this regulation is governed by a molecular mechanism which includes ADP-sensitive binding of PKA to the Kir6.2 subunit at serine residue S372 or to the SUR1 subunits of K_ATP_ channels at serine residues S1571 (human only) and S1448 [82,83].

Inhibition of K_ATP_ channels can also be mediated by Epac2A [84]. Some 20 years ago, in vitro studies demonstrated that Epac2A interacts with the nucleotide-binding fold-1 (NBF-1) at the SUR1 subunit of the K_ATP_ channel [85,86,87]. Since SUR1 also binds with Kir6.2 subunit to form functional inwardly rectifying K_ATP_ channels [79], this finding suggested that Epac2A might serve as an accessory subunit of the K_ATP_ channel. The interaction of Epac2A and SUR1 may explain why cAMP elevating agents (e.g., forskolin, glucagon) inhibit K_ATP_ channel functioning. Indeed, the study by Kang et al. confirmed that, in human β cells and rat insulin-secreting INS-1 cell line, cAMP analogues selective for the Epac2A pathway inhibit the functioning of K_ATP_ channels [84]. This regulation by Epac2A is of great interest since it was reported that SUs can directly activate Epac2A and that activation of Epac2A signaling is required for SU-induced insulin secretion [33,45,46].

#### 2.1.2. K_v_ Channels

In human beta cells, the voltage-dependent K^+^ current is mediated by delayed rectifying K^+^ (K_v_) channels and big K^+^ conductance (BK) channels [88], which are involved in the repolarization phase of action potentials [89]. Among the K_v_ channels, K_v_2.1 channels are an important component of the delayed rectifying current identified in mammalian beta cells [90,91]. However, there are substantial differences in ion channel subtypes among species that shape the action potentials [5]. In mice, the most widespread channels are the K_v_2.1 [88], whereas, in men, single cell transcriptome profiling revealed that the expression of KCNB2 gene, which encodes K_v_2.2, is nearly 10-fold higher than the expression of KCNB1 encoding K_v_2.1, suggesting a dominant role of K_v_2.2. in human beta cells [92]. Together, K_v_2.1 and K_v_2.2 channels account for 65%–80% of potassium currents in human beta cells, and their inhibition with various inhibitors increases beta cell activity [89,93].

Recent studies reported that K_v_ channels are inhibited by cAMP, which results in prolongation of action potential duration, subsequently elevating intracellular Ca^2+^ induced by activation of cAMP signaling [94], and magnesium-ATP (MgATP) plays a pivotal role in maintaining the activity of K_v_2.1 channels [95]. An electrophysiological study of isolated rat beta cells reported that K^+^ currents through Kv channels are regulated by cAMP/PKA and not by the protein kinase C (PKC) pathway [96]. Additionally, the effect of a xanthine derivative, which increases cellular cyclic nucleotides and activates K^+^ channels in high glucose (KMUP-1), was reversed by the PKA inhibitor H-89, suggesting that KMUP-1 might be a promising pharmacological substance for treating insulin resistance [96].

Another mechanism regulating K_v_ channels was described to work through incretin stimulation of PKA. Kim et al. reported that the incretin hormone GIP suppressed ionic current through K_v_1.4 channels by activating PKA, which phosphorylates the C-terminal domain of K_v_1.4 channel and mediates rapid phosphorylation-dependent endocytosis of K_v_1.4. This mechanism highlights an important novel role for GIP in regulating surface expression of K_v_1.4 channels and modulation of potassium currents [97]. Since K_v_ channels are responsible for the repolarization phase of action potentials [89], endocytosis of Kv1.4 channels would result in slower decrease of membrane potential, thus further prolonging insulin secretion.

Many other delayed rectifying K_v_ channels are present in beta cells and may have roles other than mediating the K^+^ efflux. Specifically, K_v_2.1 channels were reported to directly facilitate insulin secretion [98] through interaction with Syntaxin-1A and SNAP-25 in the SNARE complex of the exocytotic machinery [99,100].

#### 2.1.3. TRP Channels

Auxilliary depolarizing or “leak” currents are mainly carried by influx of Na^+^ ions through transient receptor potential (TRP) channels, of which beta cells express many subtypes. Some of them are also permeable to Ca^2+^ (e.g., TRPA1, TRPC1, TRPC4, TRPM2, TRPM3, TRPM5, TRPV1, TRPV2, and TRPV4). Each TRP channel consist of four subunits which may not be of the same type, giving rise to a variety of ion channels that are regulated through various mechanisms [101]. For many TRP channels, their main roles are known, but not for all of them, and even less is known about the mechanisms that regulate their gating, selectivity, and interactions. Only those with known regulatory mechanism in beta cells are briefly presented here.

TRPM2 channels are expressed on cell membranes of rodent and human beta cells as well as in many cell lines and are involved in insulin secretion in response to glucose stimulation [102,103]. Their most potent activator is cyclic ADP-ribose (cADPR) [104], but the channel opening also requires binding of Ca^2+^ to the transmembrane domains [105,106]. Other activators include nitric oxide, H_2_O_2_, free radicals, and β-NAD^+^. TRPM2 was shown to activate with nanomolar concentrations of GLP-1 through the cAMP-Epac2A pathway [103,107,108], but the involvment of cADPR and a glucose stimulus are needed [109]. Contrarily, inhibition of cAMP signaling was achieved by low concentration of adrenaline, which activates α2A adrenoceptors [110], by nanomolar ghrelin concentrations [111], and by low pH [112]. Yoshida et al. showed that activation of nonselective cation currents through TRPM2 channels by glucose and GLP-1 facilitates membrane depolarization and elevates cAMP concentration, which activates the Epac2A-mediated pathway. In addition, nonselective cation currents were attenuated in TRPM2-deficient mice and were not activated in the presence of PKA activators, supporting the stimulatory role of Epac2A and not PKA in the regulation of TRPM2 [108].

To summarize, the most prominent effect of cAMP/Epac2A/PKA pathway on plasma membrane channels and the ionic fluxes which regulate insulin secretion is the inhibition of K_ATP_ channels by Epac2A, the inhibition of K_V_ channels by cAMP, and the K_V_1.4 endocytosis.

### 2.2. The Effect of cAMP on [Ca^2+^]_IC_

The effect of GLP-1 on [Ca^2+^]_IC_ (Figure 2B) was recorded in rodent as well as human beta cells [113,114,115,116]. In low glucose, which does not increase [Ca^2+^]_IC_ per se, cAMP has almost no effect on insulin secretion [40,41], and cAMP-elevating agents are unable to raise [Ca^2+^]_IC_ in the absence of stimulatory glucose concentration [109,117,118]. This indicates that the amplifying effect of GLP-1 requires interaction with the triggering [Ca^2+^]_IC_ signal. GLP-1 increases voltage-dependent Ca^2+^ currents by increasing the activity of L-type VDCCs, and the inhibition of said channels inhibits [Ca^2+^]_IC_ rise in response to GLP-1 or forskolin [119,120,121,122]. Increased Ca^2+^ influx through L-type VDCC is due to (i) a leftward shift in voltage dependent activation and (ii) rightward shift in voltage dependent steady-state inactivation. A leftward shift in voltage dependent activation means that the maximal current through the channels is recorded at a lower membrane potential when GLP-1 is added. This is likely mediated by a PKA-induced phosphorylation of the channels. Due to a rightward shift in steady-state inactivation curve, fewer channels are inactivated at a given membrane potential and are therefore ready to be activated once again at a higher membrane potential [123,124].

Of the two L-VDCC subtypes expressed in beta cells, both can mediate the GLP-1-induced potentiation of glucose-induced insulin secretion, however, Ca_v_1.3 seems to be preferentially coupled to GLP-1 receptor activation. This signaling pathway depends on intact intracellular stores, PKA and PKC activation [125]. While both Epac2A and PKA are important in regulating the activity of Cav1.2, Cav1.3 activation is mainly a PKA-dependent process, in which Epac2A has a secondary role [125,126].

The effect of cAMP on beta cell Ca^2+^ signaling is not restricted to the voltage-dependent Ca^2+^ influx but also involves PKA-dependent and PKA-independent Ca^2+^ mobilization from internal stores (Figure 2B) [122,127,128,129]. Following beta cell depolarization and activation of VDCCs, Ca^2+^ entering cytoplasm triggers additional release of Ca^2+^ from intracellular compartments [130]. The role of this process, known as Ca^2+^-induced Ca^2+^-release (CICR), in pancreatic beta cells is still incompletely understood, especially in terms of GSIS. Since thapsigargin failed to reduce insulin secretion, it was previously believed that CICR was of minor physiological importance in GSIS [131]. With utilization of Ca^2+^ indicators with enhanced sensitivity and high-resolution Ca^2+^ imaging, the latest research suggests that, even under glucose stimulation, intracellular Ca^2+^ stores play a crucial role in shaping beta cell Ca^2+^ responses [132]. Contrary to that, it is agreed that exendin-4, a high affinity GLP-1 agonist, facilitates CICR and that this mechanism importantly contributes to beta cell Ca^2+^ response [133,134,135].

To examine how cAMP elevating agents affect CICR in beta cells, a UV flash photolysis was used to rapidly uncage Ca^2+^ from NP-EGTA, a photolabile Ca^2+^ chelator. The uncaging of Ca^2+^ generated an increase in [Ca^2+^] but did not induce CICR. The addition of exendin-4 failed to induce [Ca^2+^]_IC_ increase in beta cells before uncaging of Ca^2+^ by UV flash photolysis, indicating the effect of exendin-4 in this experiment was not mediated through membrane depolarization and subsequent opening of VDCC [133] but by sensitizing the intracellular Ca^2+^ release mechanism to the stimulatory effect of an increase in [Ca^2+^]_IC_, thereby allowing CICR to be triggered by uncaging of Ca^2+^ [129].

Several conflicting results were published regarding the role of PKA and Epac2A in modulation of beta cell internal Ca^2+^ stores, and molecular mechanisms remain obscure. Facilitation of CICR after exendin-4 stimulation was diminished but not abrogated in Epac2A KO islets, and PKA inhibition failed to (completely) prevent incretin-induced CICR in control islets [109,133,136,137]. While some studies show both PKA and Epac2A selective agonists can induce a rapid and sustained Ca^2+^ rise [109] or trigger CICR [129], other only recorded this response after PKA activation but not after application of Epac2A activators [138]. Taken together, it seems that both PKA-dependent and PKA-independent mechanisms contribute to cAMP-mediated release of intracellular Ca^2+^ stores. It is also worth pointing out that, while Ca^2+^ response remained unaffected, the inhibition of PKA nearly abolished the potentiating effect of Epac2A on glucose-induced insulin secretion, indicating that PKA has a permissive role operative further downstream in the stimulus–secretion coupling cascade [122]. Since at least in vitro half maximal activations of PKA and Epac2A occur at different cAMP concentrations, the amount of cAMP synthesized in response to selected stimulating protocols could also contribute to variability of results obtained in different studies [134].

Most studies recognize the endoplasmic reticulum as the principal intracellular Ca^2+^ source. Depletion of the ER by thapsigargin, a potent inhibitor of sarco/endoplasmic reticulum Ca^2+^-ATPases (SERCA), typically deems beta cells unresponsive to GLP-1 as well as PKA and Epac2A-selective agonists [121,133,139]. However, mobilizing Ca^2+^ from ER stores is not the only feasible way to increase [Ca^2+^]_IC_. There is a report indicating that Epac2A activators might signal through nicotinic acid adenine dinucleotide phosphate (NAADP) receptors, known also as two-pore Ca^2+^ channels, to mobilize Ca^2+^ from non-ER compartments. These acidic stores are presumed to be responsible for the fast, transient phase of Ca^2+^ response [109]. However, other studies found these non-ER stores to be less likely to play a role in GLP-1 induced CICR [133].

Whether CICR is mediated via inositol trisphosphate receptors (IP3R) or ryanodine receptors (RYR) is still debatable. In beta cells, functional receptors of both types are present, with IP3R type 3 and RYR type 2 being the most abundantly expressed [121,140]. Some argue that PKA-mediated CICR via IP3 is the major mechanism by which cAMP amplifies insulin release [138]. The effect of Epac2A was also found to be associated with IP3R, secondary to Epac2A/Rap1-mediated activation of phospholipase C-ε (Figure 2B) [133,141]. Others, however, reported that the Ca^2+^ rise in response to Epac2A-selective agonists was not accompanied by a detectable increase in IP3 and that pretreatment of cells with ryanodine or ruthenium red, antagonists of RYR, effectively blocked the Ca^2+^ response to incretins [116,121,137]. Additionally, the IP3R inhibitor xestospongin C did not affect the GLP-1 induced Ca^2+^ responses [84,109,142]. While opinions differ regarding the type of receptor, IP3 or RYR, it is generally accepted that the mobilization of intracellular stores induced by GLP-1 is cAMP-dependent [143] and acts via receptor sensitization [138]. Recent research shows that both types of receptors are important, at least in glucose-induced Ca^2+^ signaling [132].

cAMP also affects [Ca^2+^]_IC_ oscillations and other types of cellular responses, but a detailed characterization of changes in [Ca^2+^]_IC_ dynamics during metabolic amplification by cAMP is lacking in published literature. The qualitative and the quantitate diversity of results arise from the variety of methods and protocols used to investigate Ca^2+^ responses in pancreatic beta cells.

While both slow and fast [Ca^2+^]_IC_ oscillations rely on periodic entry of Ca^2+^, the fast pattern also seems to depend on mobilization of intracellular Ca^2+^ stores, and agents that increase cAMP promote the appearance of regular fast [Ca^2+^]_c_ oscillations [132,144].

Older studies, where GLP-1 was applied in pulses ranging from 30 s [121] to a few minutes, typically report a rapid [Ca^2+^]_IC_ peak, occasionally followed by a smaller, sustained [Ca^2+^]_IC_ elevation [114,121]. Holz et al. described two main temporal components of the GLP-1-induced [Ca^2+^]_IC_ dynamics and proposed a mechanism behind them. The first one is transient on the timescale of seconds and is believed to be due to CICR activated by a Ca^2+^ influx trough L-type VDCCs. The second, more sustained Ca^2+^ rise measured in minutes reflects an increased flux of Ca^2+^ ions through plasma membrane through L-type VDCCs as well as through nonselective cation channels. These fast Ca^2+^ transients were recorded in beta cells voltage-clamped at 50 mV, a membrane potential above the resting potential, to allow GLP-1 to promote the opening of VDCCs, and the rise in Ca^2+^ was not associated with a change in membrane current. However, the fast transients were blocked by nimodipine, exposure to a Ca^2+^-free medium, hypoglycemic glucose concentrations, thapsigargin, and ryanodine, all supporting the model where GLP-1 facilitates Ca^2+^ influx and RYR-mediated CICR to amplify insulin granule exocytosis [121]. Contrary to this, Kim et al. showed that only the late/sustained phase of Ca^2+^ signal is sensitive to thapsigargin and ryanodine, while the initial, transient part is thapsigargin-resistant and is believed to represent Ca^2+^ release from acidic stores [109].

When [Ca^2+^]_IC_ oscillations are recorded in response to glucose stimulation, addition of GLP-1 or cAMP agonists typically increases their frequency [115,145,146,147]. A more substantial rise in [cAMP]_IC_ achieved by simultaneous activation of AC and inhibition of PDEs (IBMX) leads to a sustained, non-oscillatory increase in [Ca^2+^]_IC_, which is sometimes preceded by an escalation in oscillatory frequency [115]. This could indicate that both cAMP concentration and dynamics are relevant for the oscillatory character of Ca^2+^ responses. cAMP elevations in beta cell were also found to be oscillatory in response to both glucose and neurohormonal amplifiers, i.e., GLP-1. [78,148]. GLP-1 induced cAMP oscillations are synchronized with Ca^2+^ responses and are mirrored by changes in PKA and Epac2A activity [43,149]. Moreover, they are coupled with pulsatile insulin secretion [150]. While many aspects of this cAMP dynamic remain unclear, it seems that the brief elevations in cAMP help contain the effects of cAMP in specific, spatially limited cellular subdomains, affecting only some of the ion channels or the exocytotic machinery adjacent to these domains [43]. Additionally, various cellular subdomains could have different oscillatory patterns and concentrations of cAMP, and this especially discreet camp pool could affect whole cell Ca^2+^ oscillations and potentially insulin secretion [149]. In some older studies, the addition of GLP-1 during glucose stimulation of mouse islets caused either additional [Ca^2+^]_IC_ elevation with attenuation or abolition of Ca^2+^ oscillations [136] or lengthening of the oscillations or a sustained peak [151]. These results as well as the ones above only describing transient and/or sustained elevation could be explained by technical limitations in measuring [Ca^2+^]_IC_. The older studies of Bode et al. and Flamez et al. used Fura-2 and Fure-PE3/AM, respectively, capturing emitted fluorescence by a CCD camera. Contemporary Ca^2+^ indicators that are compatible with confocal microscopy and have an improved sensitivity to rapid changes in Ca^2+^ dynamics offer enhanced insight into beta cell dynamics that was previously inaccessible [152].

To conclude, cAMP promotes Ca^2+^ influx through voltage dependent Ca^2+^ channels as well as its mobilization from intracellular stores. While Epac2A and PKA seem to be relevant for both mechanisms, precise molecular mechanisms remain uncertain. It is worth emphasizing that, while the changes in [Ca^2+^]_IC_ are minor, the amount of insulin secreted in response to cAMP elevation is much greater, indicating that the rise in [Ca^2+^]_IC_ is just one and probably not the most quantitatively important cAMP-sensitive step in reference [115].

### 2.3. The Effect of cAMP on Exocytosis

Distally to the triggering increase in [Ca^2+^]_IC_ in the SSC cascade, cAMP also directly affects exocytosis (Figure 2C) [127,153]. Total internal reflection fluorescencemicroscopy revealed that cAMP in the presence of glucose but not by itself enhances the frequency of fusion events of insulin granules during both phases of insulin secretion [45]. This action seems to consist of both a PKA-dependent and a PKA-independent mechanism. The latter, involving activation of Rap1 by Epac2A, is essential primarily in the first phase of insulin secretion, not by triggering exocytosis per se but by facilitating the recruitment of the granules to the plasma membrane (Figure 2C) [45]. Full understanding of mechanisms by which cAMP regulates recruitment of granules via PKA and Epac2A is not yet known, however, a Golgi-derived microtubule nucleation process [154] as well as a microtubule-associated protein termed syntabulin [155] that are both regulated by Epac2A may play a role. A recent study on Epac2A KO mice showed an attenuation of the first phase of insulin secretion, while the following second phase involved PKA signaling [45]. Some other studies reported the involvement of Epac2A also in the second phase of insulin secretion [45,122,156]. cAMP elevation was shown to stimulate Epac2A clustering at the site of docked granules, facilitating granule priming and exocytosis [157]. Epac2A/Rap signaling augments insulin secretion by increasing the size of the readily-releasable pool (RRP) of granules and by recruiting insulin granules to the plasma membrane [45]. These effects are not all Rap mediated, since Epac2A also interacts with Piccolo, Rim2α, and Rab3, all required for cAMP-regulated insulin granule exocytosis (Figure 2C) [86]. Epac2A, by complexing with the Rab3-interacting molecule Rim2, facilitates docking and priming of secretory granules [158]. Furthermore, granule acidification, a process important in granule priming, is influenced by Epac2A as well, as it regulates Cl^−^ influx [159]. Epac2A also facilitates exocytosis of already docked and primed granules [159,160], at least partially through interactions with the t-SNARE component SNAP-25 via the cAMP-Epac2A\Rim2 pathway, which is essential for exocytosis [161]. Epac2A may also regulate exocytosis as a part of the Rim2-Munc13-1-SNARE protein-syntaxin complex that results in Munc13-1 mediated unfolding of syntaxin (Figure 2C). This complex was shown to be under the control of PKA as well [162]. PKA-dependent mechanism is also involved in exocytosis by sensitizing the secretory machinery to Ca^2+^ [163], increasing the number of highly Ca^2+^-sensitive pool (HCSP) of granules [164,165] and increasing mobility and replenishment of the RRP and is therefore mainly responsible for the second phase of insulin secretion [160]. Along with the above mentioned syntaxin, SNAP-25 [161] and Snapin [49] appear to be exocytotic machinery proteins under dual control of both the PKA-dependent and the PKA-independent pathways (Figure 2C). PKA-dependent phosphorylation of Snapin thus appears to play a role in the merger of both cAMP stimulated pathways as well as in facilitation of exocytotic protein interactions and subsequent GSIS [49]. Taken together, these studies show that PKA and Epac2A, while not responsible for the triggering of exocytosis, contribute meaningfully to trafficking of insulin granules and facilitate their fusion with the plasma membrane.

## 3. The Role of cAMP in Intercellular Coupling

The pancreatic islets of Langerhans are multicellular micro-organs within which communication among a variety of cells with unique functions must occur to ensure proper control of metabolic homeostasis. The beta cells are the most abundant cell type within the islets, which, along with glucagon-producing α-cells, somatostatin-producing δ-cells, PP cells, and a minority of other cell types, constitute the endocrine component of the pancreas [166]. Islet cells communicate via direct electrical coupling through gap-junctions as well as by paracrine, autocrine, and juxtacrine signaling [52,54,167,168]. For beta cells, intercellular coupling established through gap junctions composed of Cx36 is particularly important, as it provides the necessary and the most important substrate for coordinated responses of the beta cell population, a prerequisite for the well-regulated secretion of insulin [57,62,72]. Specifically, gap junctions are a type of specialized membrane contacts that enable direct communication by allowing current-carrying ions and other small molecules to pass directly into the cytoplasm of adjacent cells, giving rise to propagating intercellular waves [5,55,56,57,58,169,170,171,172,173] [70,174,175,176,177,178]. Cx36 gap junction channels are size- and charge-selective and favor the exchange of positively charged molecules at the expense of anionic molecules [179,180]. However, the cationic selectivity of Cx36 gap junctions is not absolute, as beta cells are able to exchange negatively charged molecules, such as phosphorylated glucose metabolites and nucleotides [52,181]. While the ionic coupling is the key intercellular mediator and extends to large regions of the islets, there is increasing evidence that metabolic coupling also plays a role in intra-islet communication but is more confined and encompasses smaller clusters of few beta cells [182,183,184,185]. All the compound coupling mechanisms along with beta cell heterogeneity underlie the complex functional connectivity patterns that are usually extracted from the recorded multicellular Ca^2+^ activity [70,174,175,176,177,178]. Noteworthy, disruptions of gap-junctional communication were found to impair synchronized beta cell responses and lead to dysregulated plasma insulin oscillations and to glucose intolerance [59,61], as observed in numerous models of obesity and diabetes mellitus [62,63,70,72,186]. Moreover, prolonged exposure to high concentrations of glucose and fatty acids, as expected in diabetes, were found to downregulate Cx36 expression and disrupt the coherent patterns of intercellular synchronization [187,188], whereby some pharmacological agents seem to be able to at least partly repair the defective signaling pattern [70]. Gap junction coupling was also reported to be disrupted by pro-inflammatory cytokines, which also contribute to the decline in islet function during the pathogenesis of diabetes [189]. Along these lines, Cx36 gap junction coupling and its modulation are increasingly recognized as vital components in normal islet function [73,190,191] and potentially viable targets to help restore insulin secretion in diabetes [64,65,192].

How the neurohormonal signaling pathways influence gap-junctional communication and how they relate to the collective activity of beta cell networks are incompletely understood. First clues that cAMP affects cell-to-cell communication (Figure 2D) came from electrophysiological studies. Increasing intracellular cAMP concentration with forskolin in microdissected mouse islets enhanced conduction between couples of impaled beta cells by 24% [66]. In contrast to microdissection, in isolated islets, forskolin incubation failed to change the distribution of gap junction conductance of beta cells [193]. Finally, a third possible outcome of forskolin application on gap junction was reported in insulin-secreting cell lines where application of forskolin decreased Cx36 expression [187]. Despite these contradictory reports, only a few studies attempted to decipher the mechanism underlying the modulatory effect of cAMP on gap junctions. A chronic hyperlipidemia-induced down-regulation of Cx36 was reported to be associated with an overexpression of the inducible cAMP early repressor ICER-1 γ [194,195], thereby implying a direct impact of the cAMP/PKA pathway on the gap junctional coupling. Moreover, additional evidence from other tissues, such as retinal AII amacrine cells or neurons of the inferior olive, corroborated the inhibitory role of PKA on Cx36 function [68,74,196]. Conversely, in rat myocardial cells, cAMP was found to enhance Cx43-mediated gap junctional coupling through both PKA and Epac2A pathways [67,71], thereby substantiating further the complex and multifaceted role the incretin pathways play in multicellular systems.

Within the islets, the next line of evidence of the involvement of neurohormonal signaling pathways in cell-to-cell communication came from mice with ablation of Cx36. The logic behind this approach is that the incretin pathway (via PKA or Epac2A) increases insulin secretion, and if this effect is modulated in Cx36 KO mice, this would correlate the effect of either PKA or Epac2A to changes in gap junctional conduction. Along this line, the PKA specific agonist 6-Bnz-cAMP, but not the Epac2A specific agonist 8-Me-2-O-pCPT-cAMP, produced a significant elevation of insulin secretion from Cx36 KO isolated islets compared to islets with intact intercellular connectivity [69]. This suggested that the PKA arm of the incretin pathway acts via a change in gap junction conductance, an observation that was also noticed in MIN-6 cell lines but with a contradictory effect of increasing insulin secretion when PKA was activated [197]. To complicate things even further, the modulatory effect of cAMP on gap junctions was studied only at low glucose (2 mM) [69]. The authors proposed that cAMP functions to suppress insulin release only at basal blood glucose levels, and that this mechanism may serve as an alternative mechanism to the more commonly accepted path in which hyperpolarizing currents through gap junctions from less activated beta cells inhibit the more active beta cells. Furthermore, in human donor islets, by using a different methodological approach, an effect of cAMP on intercellular coupling was demonstrated by pharmacologically blocking gap junctions using 18-α-glycyrrhetinic acid that resulted in desynchronization of the calcium response to GLP-1 and decreased insulin secretion [70]. Surprisingly, inhibiting gap junctional communication did not decrease insulin secretion in response to stimulation by 11 mM glucose only, i.e., without GLP-1, pointing to a possible non-gap junctional action of gap junction blockers on the incretin signaling pathway. Moreover, it was shown that human islets respond to secretagogues in a highly coordinated manner guided by a network of interlinked cells, and lipotoxicity was shown to impair these responses. Notably, the deleterious effects of chronically elevated free fatty acid levels on the islet dynamics underlying insulin release were argued to be brought about through PKA- and cAMP-dependent inhibition of intercellular communication via gap junctions [70].

Very recently, Farnsworth et al. [73] examined the respective roles of PKA and Epac2A in beta cell-to-cell coupling very explicitly. In their study, in islets with decreased Cx36 coupling due to the action of pro-inflammatory cytokines, exendin-4, which increases cAMP, was found to overcome cytokine-induced dysfunction of islets, and restore the coupling. Their results showed that both neurohormonal signaling pathways play a role in mediating cAMP regulation of Cx36 in islets but in a different manner. PKA was suggested to regulate Cx36 coupling via fast mechanisms, such as Cx36 phosphorylation and channel gating, whereas Epac2A was reported to modulate Cx36 coupling via slower mechanisms, such as trafficking, assembly, or turnover of Cx36 channels. These concepts are presented in Figure 2D, where it is illustrated that PKA regulates Cx36 coupling via gating of gap junction channel conductance, whereas Epac2A operates on slower temporal scales by regulating Cx36 trafficking, gap junction assembly, or Cx36 endocytosis in beta cells. Further investigations will be needed to assess these ideas and the underlying mechanisms in more detail, but, in general, they go well in hand with what was reported in other systems [67,68,72,73,198].

**Figure 3:**
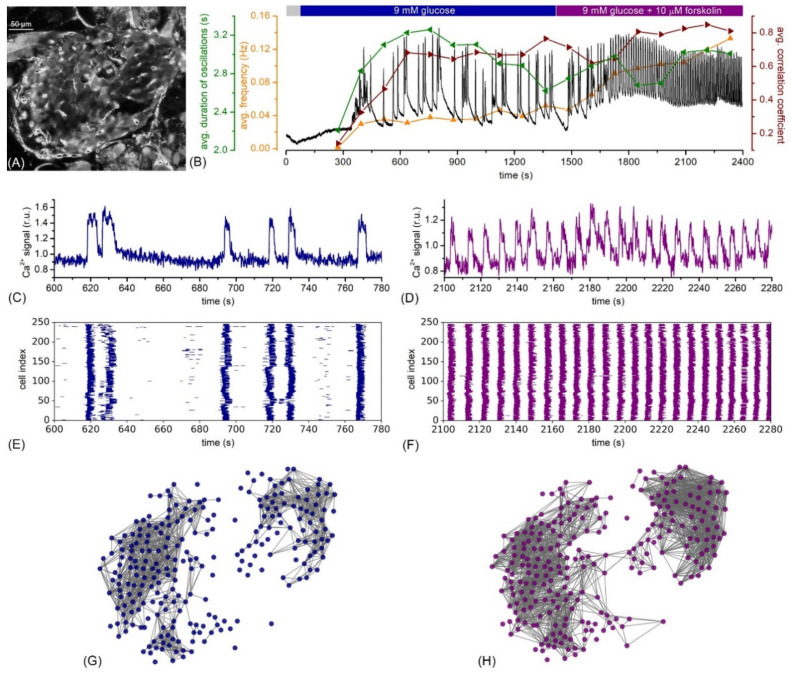
Multicellular beta cell activity under stimulatory glucose levels and the additional activation of the neurohormonal amplifying pathways by forskolin. (**A**) Confocal image of an islet of Langerhans within the tissue slice with well stained cells using fluorescent calcium dye Calbryte 520AM; (**B**) average Ca^2+^ signal calculated based on all beta cells in the islet (black line), the temporal evolution of the frequency (orange), the duration of oscillations (green), and the average correlation coefficient (red). For the calculation of signaling parameters, we used a sliding window of 3 min and a window overlap of 1 min. The stimulation protocol is visualized on the top of the panel, whereby the grey rectangle signifies substimulatory 6 mM glucose concentration; (**C**–**H**) recorded traces from an exemplary cell (**C**,**D**), raster plots of binarized Ca^2+^ activity of all selected cells in the islet (**E**,**F**), and the corresponding functional beta cell networks (**G**,**H**) under stimulation with 9 mM glucose (**C**,**E**,**G**) and with 9 mM glucose plus 10 µM forskolin (**D**,**F**,**H**). The multicellular Ca^2+^ activity of beta cells was captured by means of confocal laser scanning microscopy in an acute pancreatic tissue slice (see Refs. [58,200] for methodological details).

Finally, in our very recent study, we investigated explicitly how cAMP-mediated amplification affected the collective beta cell activity within the islets [199]. Our results showed that the activation of cAMP signaling did not only profoundly increase beta cell activity but also enhanced synchronicity and the coordination of intercellular signals. To provide a clear demonstration of this behavior, we present in Figure 3 an exemplary recording of an islet that was stimulated with 9 mM glucose and subsequently with 10 µM forskolin. The increase in cytosolic cAMP caused by forskolin evoked a more than a two-fold increase in the oscillation frequency, a slight decrease in the duration of oscillations, and an increase in the level of synchronization, reflected by the average correlation coefficient. The raster plots in Figure 3E,F indicate that, after the application of forskolin, the multicellular dynamics were dominated by global and well-aligned Ca^2+^ waves, while under stimulation with glucose only, the activity patterns were more erratic. To assess the collective activity in further detail, we constructed functional connectivity networks [178] separately for the stimulation with glucose only and glucose with additional forskolin. The results presented in Figure 3G,H indicate that increased levels of cAMP led to a denser and a more integral network (Figure 3H), thereby signifying a higher degree of synchronization between beta cells when compared to glucose stimulation only (Figure 3G). This is well in agreement with our previous results, where, in control experiments with prolonged glucose stimulation only, synchronized behavior diminished with time, but the activation of cAMP prevented this effect and enhanced the degree of beta cell synchronicity [199]. Most importantly, performing the same set of experiments with Epac2A KO mice, stimulation with forskolin restored the functional network integrity only partly. These observations imply that both arms of the neurohormonal amplifying pathway play a role by shaping the collective beta cell activity in islets and parallel in part with the recent findings by Farnsworth et al. [166], where increased cAMP was also identified as a promotor for intercellular coupling. Moreover, the absence of Epac2A, which might be associated with impaired connexon trafficking pathways [166], apparently hinders the beta cells from Epac2A KO mice to fully exploit the positive effects that increased cAMP has on the beta cell network activity. Nevertheless, the precise roles that the different arms of the neurohormonal signaling pathway play in the functionality of beta cell networks and the question of to what extent the effect of cAMP on insulin secretion can be attributed to its impact on intercellular coupling remain to be elucidated in future studies [73].

## 4. The Role of cAMP during the Development of Insulin Resistance

In insulin resistance, compensatory beta cell adaptation ensures increased insulin secretion, which is usually able to sustain normoglycemia for long periods of time [201,202]. However, when beta cell compensation fails to meet increasing insulin demands imposed by insulin resistance, glucose intolerance first becomes apparent as mild and later overt hyperglycemia that characterizes a full-blown T2DM [203]. Several high-quality studies established that the elevated plasma insulin levels in complete compensation (hyperinsulinemia and normoglycemia) or in partially compensated prediabetic state (hyperinsulinemia and mild hyperglycemia) are related with morphological adaptation in the form of an increased beta cell mass [204]. Importantly, the evidence is not unanimous, and some reports reported only moderate [205] or no increases in beta cell mass in insulin resistance compared with controls [206]. It was demonstrated that GLP-1 enhances beta cell survival by activating beta cell proliferation and differentiation and inhibiting apoptosis. The underlying mechanism is relatively complex, involving a PKA-mediated translocation of a protein complex composed of a transducer of regulated CREB activity-2 (TORC2) and a cAMP-response element binding protein (CREB) into the nucleus, where it induces transcription of genes that are involved in beta cell survival. It remains to be clarified whether this is solely a PKA-mediated effect [207]. The GLP-1 enhancing effect was indirectly corroborated in an animal model of insulin resistance, where GLP-1 treatment increased the amount of insulin secreted following a glucose load [208] (for review on (dis)advantages of chemically inducing insulin resistance in animal models, see [209]). Importantly, later, during worsening of insulin resistance and glucose intolerance, beta cell mass was typically decreased [210]. In a diet-induced mouse model of diabetes supplemented with streptozotocin (STZ) administration, chronic GLP-1R stimulation partially rescued the decrease in beta cell mass [211], confirming the role of cAMP in morphological adaptation, even in the setting of decompensated diabetes.

In stark contrast, comparatively few studies addressed the role of beta cell functional adaptation in the compensatory response to insulin resistance, and thus changes in beta cell SSC, and intercellular coupling remain poorly characterized in obesity [212,213,214]. Additionally, most data about beta cell functional adaptation come from isolated beta cells [213,214], which are unable to fully capture the normal physiological scenario compared with isolated islets [215,216] or tissue slices [58,171]. In a genetic model of obesity (*ob/ob*) characterized by hyperinsulinemia and moderate hyperglycemia, beta cells displayed enhanced mitochondrial function and electrical activity, left shift in sensitivity and a preponderance of slow [Ca^2+^]_IC_ oscillations, increased GSIS, and reduction in beta cell coupling [188]. Similarly, in high fat diet (HFD)-induced insulin resistant mice that were hyperinsulinemic and normoglycemic, insulin gene expression and GSIS were increased, and glucose-induced [Ca^2+^]_IC_ and exocytotic responses were enhanced [217]. Similarly to the decrease in beta cell mass, beta cell function starts to deteriorate once overt type 2 diabetes mellitus develops. Beta cell decompensation encompasses oxidative and endoplasmic reticulum (ER) stress, dedifferentiation, and lower expression of GLUT2, glucokinase, as well as anomalies in ER Ca^2+^ mobilization. Additionally, beta cell connectivity was shown to deteriorate during progression of T2DM, and this might be one of the earliest defects [65,72,203]. These alterations are believed to be a consequence of chronic hyperglycemia, hyperlipidemia, and a proinflammatory metabolic state [64,70,177,189,218].

Limited data are available to explain which aspects of beta cell functional adaptation depend on the GLP-1 receptor signaling cascade, cAMP, and specifically on PKA and Epac2A. Epac2A seems to play a role in potentiation of GSIS under conditions of increased demand for insulin, e.g., in insulin resistance, such as during obesity [219]. Studies employing HFD in the Epac2A KO mice to induce insulin resistance implicated that Epac2A ablation results in increased body weight, impaired glucose tolerance [50,51], and decreased GSIS during 4 weeks of a HFD diet intervention compared with control mice on the same diet [50]. Surprisingly, another study reported an increase in GSIS during 8 weeks of HFD diet in Epac2A KO mice compared with control mice on the same diet [51]. A mechanistic explanation for how Epac2A could bring about an improvement in GSIS during an ipGTT in insulin resistance is not available at the moment, but it was suggested that, under these conditions, glucose-induced production of cAMP and Epac2A activation might become operative [220]. However, experimental verification is missing as well as an explanation for the differential effect on GSIS in Epac2A KO mice on HFD at 4 and 8 weeks. To complicate matters even further, it was demonstrated that the Epac2A-dependent GSIS potentiation also involves the brain–islet axis. The neuropeptide Orexin A secreted from the lateral hypothalamic area potentiated GSIS in vitro, and this effect was blocked in Epac2A-deficient mice [221]. Paradoxically, the plasma levels of OXA were decreased in rodent models of T2DM [222,223] and in obese women [224]. To the best of our knowledge, a single study addressed the role of PKA in the setting of insulin resistance. Disruption of the protein PKA inhibitor beta (PKIB), an effective inhibitor of PKA activity, improved glucose sensitivity and GSIS in a 20-week HFD-induced mouse model [225], suggesting a PKA-potentiating effect on GSIS. To our knowledge, there are no data on the roles of Epac2A and PKA in changes of [Ca^2+^]_IC_ oscillations, sensitivity of the exocytotic machinery, or intercellular coupling during diet-induced T2DM. A single study demonstrated that, in human islets, cAMP stimulates synchronous [Ca^2+^]_IC_ increases that augment insulin secretion, and the signals become asynchronous with an accompanying reduction in insulin secretion in lipotoxic conditions [70].

Finally, a role of Epac2A was implicated in hypersecretion of insulin observed in combination therapies for T2DM treatment [226]. Coadministration of liraglutide (a GLP-1 R agonist) and glimepiride (SU) resulted in increased blood glucose during OGTT in Epac2A KO mice on HFD. These results indicate that the glucose-lowering effect of the combination of liraglutide and glimepiride is diminished in Epac2A KO mice. Thus, Epac2A seems to play a role in insulin secretion induced by the combination of an incretin and SU, especially in a model of diet-induced obesity, and may provide an explanation for the SU-dependent difference in the incidence rate of hypoglycemia observed in combination therapies. In sum, while cAMP tends to have a positive role in beta cell morphological and functional adaptation to insulin resistance, the relative lack of data in this important field warrants further studies to help us understand the underlying mechanisms.

## 5. Conclusions

Concentrating on a possible role of cAMP in general and its two main signaling arms in specific, our journey led us from the most proximal membrane potential step in the SSC cascade, via the central [Ca^2+^]_IC_ changes, to the most distal event, exocytosis. We also addressed the aspect of intercellular coupling and the relevance in vivo. It is quite self-evident that our knowledge about the role of cAMP is limited and biased due to the influence of historically and currently available electro- and optophysiological tools, pharmacological agents, development of specific animal models, and access to human tissue, among many other factors. Since a deeper insight into the normal intra- and intercellular coupling in beta cells and the role played by cAMP is central for a better understanding of the natural history of diabetes mellitus as well as for prevention, finding new targets, and developing new pharmacological approaches, we firmly believe that future studies will help push the frontiers of knowledge and truly hope that our review helped identify some most needed next steps.
